# Influence of ICU-bed availability on ICU admission decisions

**DOI:** 10.1186/s13613-015-0099-z

**Published:** 2015-12-30

**Authors:** René Robert, Rémi Coudroy, Stéphanie Ragot, Olivier Lesieur, Isabelle Runge, Vincent Souday, Arnaud Desachy, Jean-Paul Gouello, Michel Hira, Mouldi Hamrouni, Jean Reignier

**Affiliations:** Réanimation Médicale, Université de Poitiers, CHU Poitiers, Inserm Unit CIC 1402; Groupe ALIVE, Poitiers, France; Réanimation Polyvalente, Centre Hospitalier La Rochelle, La Rochelle, France; Réanimation Polyvalente, Centre Hospitalier Angoulême, Angouleme, France; Réanimation Médicale, Université D’Angers, CHU Angers, Angers, France; Medical-Surgical Intensive Care Unit, Hospital Center, 45067 Orleans, France; Surgical Intensive Care, District Hospital, 35400 Saint-Malo, France; Medical-Surgical Intensive Care, District Hospital, 36000 Chateauroux, France; Medical-Surgical Intensive Care, District Hospital, 28018 Chartres, France; Medical Intensive Care, University of Nantes, CHU Nantes, Nantes, France

**Keywords:** Admission, ICU, Triage, Scarcity, Refusal, Bed occupancy

## Abstract

**Background:**

The potential influence of bed availability on triage to intensive care unit (ICU) admission is among the factors that may influence the ideal ratio of ICU beds to population: thus, high bed availability (HBA) may result in the admission of patients too well or too sick to benefit, whereas bed scarcity may result in refusal of patients likely to benefit from ICU admission.

**Methods:**

Characteristics and outcomes of patient admitted in four ICUs with usual HBA, defined by admission refusal rate less than 11 % because of bed unavailability, were compared to patients admitted in six ICUs with usual low bed availability (LBA), i.e., an admission refusal rate higher than 10 % during a 90-day period.

**Results:**

Over the 90 days, the mean number of days with no bed available was 30 ± 16 in HBA units versus 48 ± 21 in LBA units (*p* < 0.01). The proportion of admitted patients was significantly higher in the HBA (80.1 %; *n* = 659/823) than in the LBA units [61.6 %: *n* = 480/779; (*p* < 0.0001)]. The proportion of patients deemed too sick to benefit from admission was higher in LBA (9.0 %; *n* = 70) than in the HBA (6.3 %; *n* = 52) units (*p* < 0.05). The HBA group had a significantly greater proportion of patients younger than 40 years of age (22.5 %; *n* = 148 versus 14 %; *n* = 67 in LBA group; *p* < 0.001) and higher proportions of patients with either high or low simplified acute physiologic score II values.

**Conclusions:**

Bed availability affected triage decisions. Units with HBA trend to admit patients too sick or too well to benefit.

## Background

Intensive care unit (ICU) bed availability varies considerably throughout the world [1, 2] and within individual countries, including France [3, 4]. The demand for ICU beds is expected to increase due to the growth and aging of the population, increasing long-term survival of patients with chronic diseases associated with episodes of acute illness, and changing perceptions about the profile of patients likely to benefit from ICU admission [1, 2]. The ideal ICU bed/population ratio is that capable of ensuring that all patients likely to benefit from critical care can be admitted to the ICU, while keeping bed occupancy high, as unoccupied beds result in costs for no benefit. One factor that may influence this ratio is the set of criteria used by intensivists to triage patients to ICU admission, which should ensure that patients too well or too sick to benefit from critical care are not admitted [5, 6]. However, in everyday practice compliance with recommendations for ICU triage is low [7]. The problem is not simply one of demand, as the criteria used to define demand vary across ICUs and over time within ICUs. Several studies showed that triage was influenced by factors unrelated to the patients, such as bed availability [8–12]. To date, research in this field has focused on the influence of bed unavailability on triage decisions and has shown that bed scarcity is associated with fewer admissions and greater acute-illness severity in admitted patients [10, 13, 14]. In addition, patients admitted at times of bed shortage had shorter stays, were sicker at discharge than patients admitted at times of greater bed availability, and may have a greater risk of early readmission [14, 15]. ICU bed scarcity was associated with higher refusal rates of patients considered too sick to benefit or requiring palliative care [12, 16] and to higher frequency of decisions to forego life-sustaining treatment [16].

Few data are available on ICU admission patterns in units with high bed availability (HBA), and therefore, low rates of refusal to admit referred patients. An advantage of HBA is the ability to admit all patients likely to benefit, but a concomitant risk is that patients too sick or too well to benefit might be admitted, thus resulting in unnecessary suffering for the patients and in increased healthcare costs.

Here, our purpose was to assess the potential influence of ICU-bed availability on the clinical characteristics of the patients admitted and refused for ICU admission.

## Patients and methods

We used data from a previous study by our group that compared outcomes of patients admitted to the ICU at first referral to those of patients who were denied ICU admission only because the unit was full [17]. For the present companion study, we focused on the patients who were admitted at first referral. We divided the ten participating ICUs into two groups based on mean bed availability over two 45-day periods constituting the 90-day study period, above or below the median 10 % value of admission refusal because the unit was full. Thus, the two groups were as follows: low bed availability (LBA) greater than 10 % rate of admission refusals due only to a full unit and HBA as a refusal rate for a full unit no greater than 10 %. The start and the end of each period were identical for all centers. We did not include patients referred to intermediate care units, transferred from one ICU to another based on a need for specific resources, or previously refused admission to another ICU.

Every day, it was reported if there was at least one time during the day in which no bed was available for a new admission. The numbers of patients who were denied ICU admission because they were deemed too sick or too well to benefit was documented prospectively as a reason for refusal ICU-admission. None of the study ICUs had a standardized protocol for triage to ICU admission, but the policy in all ten participating ICUs was to base admission decisions on published guidelines [5, 6]. As in the true life practice, the criteria used to characterize a patient too sick or too well to benefit were left to the senior physician in charge of ICU admission decision. None of the participating centers had a specific mobile emergency team and only two participating centers (one in each group) had distinct closed intermediate care units which were not involved in the study. None of the participating center had palliative care unit able to admit patients in emergency condition.

### Data collection

At ICU referral, the intensivist in charge of the admission decision recorded the following data: age; sex; reason for referral; main diagnosis; presence of shock, jaundice, coagulation disorders, and/or plasma creatinine level higher than 250 μmol/L; need for mechanical ventilation and/or renal replacement therapy; Glasgow Coma Scale score; and history of malignancy, chronic obstructive pulmonary disease, diabetes, cirrhosis, and/or NYHA Class IV congestive heart failure. McCabe category (non fatal, fatal within 6 months, fatal within 5 years) was used to estimate underlying prognosis.

For patients admitted to the ICU admission the following data were recorded: and SAPS II; need for, and duration of, mechanical ventilation; need for renal replacement therapy; and vital status at ICU discharge. Patients were followed-up for 2 months after study inclusion, and vital status was recorded on days 28 and 60; when patients were discharged from hospital, their follow-up was determined by phone call to the patient or to his personnel physician if necessary.

The study was approved by our local clinical research committee. According to French law on medical research, written informed consent from the patient or next-of-kin was not required for this observational study.

### Statistical analysis

Bivariate analyses were performed using the Student’s *t* test or Mann–Whitney *U* test for continuous variables. The *Chi*-square test, or Fisher’s exact test when appropriate, was used to compare proportions. Age was stratified into three categories arbitrarily defined: younger than 40 years, 40–75 years, and older than 75 years. SAPS II values at ICU admission were stratified into three categories also: lower than 16 (corresponding to a theoretical death prediction less than 2 %); 16–59, and higher than 59 (corresponding to a theoretical death prediction of almost 70 %).

Mortality rates at D28 and D60 were compared between the two types of units with univariate logistic regression. Moreover, factors associated with mortality were assessed by means of multivariate logistic regression analyses. The models were built using a backward manual procedure performed on a maximal model including the type of units (forced variable) and all factors that were associated with mortality with *p* < 0.05 in univariate logistic regression. Results are given as odds ratio (OR) with 95 % confidence intervals (CI).

For all analyses, a *p* value less than 0.05 was considered as significant.

## Results

**Fig. 1:**
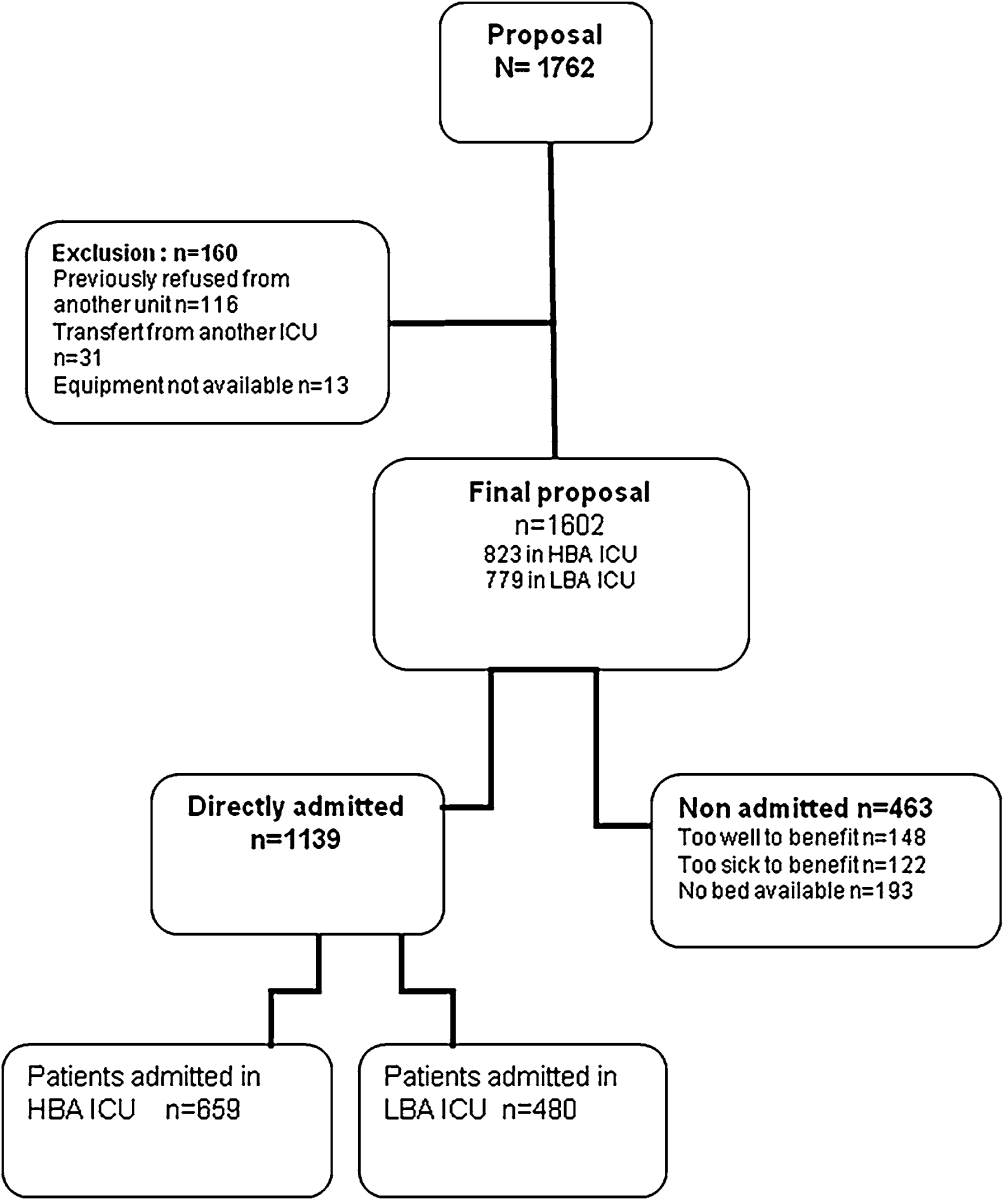
Flow chart. (*HBA ICU* high bed availability ICU with rate of refusal <11 %; *LBA ICU* low bed availability ICU with rate of refusal >10 %)

Of the 1762 patients with a first ICU referral, 160 patients did not meet selection criteria, leaving 1602 patients for the analysis (Fig. 1). Among them, 812 were referred to one of the six ICUs with HBA (in one university and five non-university hospitals) and 790 to one of the four ICUs with LBA (in one university and three non-university hospitals). The total number of ICUs beds was 65 (median, 10.8; range, 8–13) in HBA units and 66 (median, 16.5; range, 12–24) in LBA units. The ratio between the number of ICU bed and senior intensivist was identical in both groups (median = 2). Over the 90-day study period, the mean number of days with no bed available was 48 ± 21 in the 4 units with LBA versus 30 ± 16 in the 6 units with HBA (*p* < 0.01).

Of the 1602 patients, 1139 were admitted immediately to the ICU and 463 were denied admission, for the following reasons: full unit, *n* = 193; patient considered too sick to benefit, *n* = 122; and patient considered too well to benefit, *n* = 148. As expected, the proportion of admitted patients was significantly higher in the HBA (80.1 %; 659 out 823 proposals) than in the LBA units (61.6 %: 480/779 proposals; *p* < 0.0001). The proportion of patients refused admission because they were considered too sick to benefit was higher in the LBA group (9.0 %) than in the HBA group (6.3 %) (*p* < 0.05), whereas the proportions of patients refused because they were considered too well to benefit were similar in the two groups (10.3 and 8.3 %, respectively) (Table 1).

**Table 1:** Patients refused for admission

	Centers with HBA	Centers with LBA	*P*
Proposal (nb)	823	779	
Age value ± SD	57.5 ± 19.4	61.5 ± 17.6	<0.001
Too well to benefit nb (%)	68 (8.3)	80 (10.3)	0.17
Too sick to benefit nb (%)	52 (6.3)	70 (9.0)	0.04
No bed available nb (%)	44 (5.3)	149 (19.1)	<0.0001

*HBA* high bed availability, *LBA ICU* low bed availability

Characteristics of the 659 patients admitted to HBA units and of the 480 admitted to LBA units are listed in Table 2. HBA units admitted younger patients, with a significantly higher proportion of patients younger than 40 years, compared to LBA units (*p* < 0.001). The HBA group had a significantly greater proportion of patients admitted for shock, compared to the LBA group. None of the other clinical characteristics studied differed significantly between the two groups. The mean SAPSII value on admission was not significantly different between the two groups but the SAPSII value distribution differed significantly, with higher proportions of patients having low (≤15) or high (>59) values in the HBA group than in the LBA group (Table 2). In the subset of patients with SAPS ≤15, there was no difference regarding some major clinical characteristics neither before admission justifying ICU admission nor for the frequency of mechanical ventilation during ICU stay (Table 3). Mortality rates at 28 and 60 days did not differ significantly between the two groups even after adjustment on the potential confounders (Tables 4, 5).

**Table 2:** Clinical characteristics at proposal for ICU admission of the patients directly admitted to ICU with high (LBA) and low refusal rate (HBA)

	Centers with HBA *N* = 659	Centers with LBA *N* = 480	*P*
Age (mean ± SD) (years)	57 ± 19	60 ± 17	0.006
Age <40	148 (22.5)	67 (14.0)	0.001
40≤ age ≤75	509 (77.2)	302 (62.9)	
Age >75	123 (18.7)	111 (23.1)	
Sex (male/female) %	383/276	286/194	0.62
Characteristics on proposal, *n* (%)			
Shock	178 (27.3)	103 (21.7)	0.03
Catecholamine infusion	97 (15.0)	52 (11.0)	0.05
Glasgow score	11 ± 4	12 ± 4	0.09
Platelets count < 50,000/dl	11 (1.75)	13 (2.8)	0.24
Prothrombine time < 30 %	21 (3.4)	17 (3.7)	0.78
Creatinine level > 250 µmol/l	76 (12.1)	50 (10.9)	0.53
Jaundice	18 (2.8)	18 (3.8)	0.37
Mechanical ventilation (all setting)	289 (45)	189 (40)	0.10
NIV	37 (6)	32 (7)	0.55
Oxygen administration >10L/min	134 (4)	80 (17)	0.08
Preexisting diseases			
COPD	47 (7)	21 (4)	0.05
Cardiac insufficiency	34 (5)	22 (5)	0.65
Evolutive malignancy	84 (13)	65 (14)	0.69
Cirrhosis	27 (4)	30 (6)	0.10
Renal failure with ERT	16 (3)	12 (3)	0.94
Mac Cabe score			0.30
0	340 (53)	236 (50)	
1	199 (31)	166 (36)	
2	99 (16)	65 (14)	
SAPS2			
Mean ± standard deviation	41 ± 21	41 ± 20	0.77
Median (25th; 75th)	37 (25–53)	38 (27–52)	
Distribution of SAPS2 values, *n* (%)			0.004
SAPS2 ≤15	64 (9.7)	28 (5.8)	
16≤ SAPS2 ≤59	468 (71.0)	381 (79.4)	
SAPS2 >59	127 (19.2)	71 (14.8)	

*COPD* chronic obstructive pulmonary disease, *NIV* non invasive ventilation, *ERT* extra renal therapy

**Table 3:** Clinical characteristics related to in the subgroup of patients with SAPS 2 ≤ 15 admitted to ICU

	HBA *n* = 64	LBA *n* = 28
Shock prior ICU admission (%, *n*)	11, 7	3, 8
Mechanical ventilation prior ICU admission (including non invasive ventilation) (%, *n*)	16, 10	19, 5
High level oxygen administration (>10L/min) prior ICU admission (%, *n*)	8, 5	13, 4
Glasgow <12 prior ICU admission (%, *n*)	23, 15	13, 4
Mechanical ventilation during ICU stay. (%, *n*)	25, 16	33, 9

*HBA* high bed availability, *LBA* low bed availability

**Table 4:** Factors associated with mortality at D28

	Dead D28 *N* = 276	Alive D28 *N* = 860	Non adjusted analysis		Adjusted analysis	
			Crude OR	*p*	Adjusted OR	*P*
ICU						
With low refusal rate (HBA)	121 (43.8)	359 (41.7)	1	0.54	1	
With high refusal rate (LBA)	155 (56.2)	501 (58.3)	1.09 (0.83–1.43)		1.34 (0.92–1.95)	0.12
Age (mean ± SD)	67 ± 14	55 ± 19	1.04 (1.03–1.05)	<0.001		
Sex						
Male (%)	179 (65)	489 (57)	1		1	
Female (%)	97 (35)	371 (43)	0.71 (0.54–0.95)	0.02	0.66 (0.45–0.98)	0.04
Characteristics on proposal, *n* (%)						
Shock	134 (48.6)	146 (17.0)	4.73 (3.51–6.37)	<0.001	1.81 (1.21–2.72)	0.004
Catecholamine infusion	82 (29.7)	66 (7.7)	5.21 (3.63–7.48)	<0.001		
Glasgow score	9.8 ± 5.0	11.9 ± 4.1	0.90 (0.87–0.93)	<0.001		
Platelets count <50,000/dl	11 (4.0)	13 (1.5)	2.94 (1.30–6.64)	0.007		
Prothrombine time <30 %	24 (8.7)	14 (1.6)	6.17 (3.14–12.11)	<0.001	2.65 (1.09–6.44)	0.03
Creatinine level >250 µmol/l	46 (16.7)	80 (9.3)	2.19 (1.48–3.25)	<0.001		
Jaundice	14 (5.1)	22 (2.6)	2.09 (1.05–4.15)	0.03		
Mechanical ventilation (all setting)	148 (53.6)	328 (38.1)	1.93 (1.46–2.54)	<0.001		
NIV	15 (5.4)	54 (6.3)	0.87 (0.48–1.58)	0.65		
Oxygen administration >10L/min	67 (24.3)	147 (17.1)	1.59 (1.14–2.21)	0.006		
Preexisting diseases						
COPD	15 (5.4)	52 (6.0)	0.93 (0.51–1.68)	0.81		
Cardiac insufficiency	22 (8.0)	34 (3.9)	2.20 (1.26–3.84)	0.005		
Evolutive malignancy	44 (15.9)	105 (12.2)	1.42 (0.97–2.08)	0.07		
Cirrhosis	26 (9.4)	31 (3.6)	2.89 (1.68–4.97)	<0.001		
Renal failure with ERT	8 (2.9)	20 (2.3)	1.30 (0.56–2.98)	0.54		
Mac Cabe score						
0	81 (31.4)	493 (58.4)	1		1	
1	99 (38.4)	265 (31.4)	2.27 (1.64–3.16)	<0.001	1.56 (1.02–2.37)	0.04
2	78 (30.2)	86 (10.2)	5.52 (3.75–8.12)	<0.001	3.66 (2.21–6.06)	<0.001
SAPS2	62 ± 20	34 ± 16	1.08 (1.07–1.10)	<0.001	1.08 (1.06–1.09)	<0.001

*NIV* non invasive ventilation, *ERT* extra renal therapy

**Table 5:** Factors associated with mortality at D60

	Dead D60 *N* = 308	Alive D60 *N* = 823	Non adjusted analysis		Adjusted analysis	
			Crude OR	*p*	Adjusted OR	*P*
ICU						
With low refusal rate (HBA)	175 (56.8)	479 (58.2)	1	0.67	1	
With high refusal rate (LBA)	133 (43.2)	344 (41.8)	1.06 (0.81–1.38)		1.30 (0.90–1.56)	0.16
Age (mean ± SD)	67 ± 14	54 ± 19	1.05 (1.04–1.05)	<0.001		
Sex						
Male (%)	204 (66)	459 (56)	1		1	
Female (%)	104 (34)	364 (44)	0.64 (0.49–0.84)	0.002	0.54 (0.37–0.78)	0.001
Characteristics on proposal, *n* (%)						
Shock	145 (47.4)	134 (16.3)	4.70 (3.51–6.29)	<0.001	1.83 (1.23–2.72)	0.003
Catecholamine infusion	85 (27.6)	63 (7.6)	4.71 (3.29–6.75)	<0.001		
Glasgow score	10.0 ± 4.8	11.9 ± 4.1	0.91 (0.88–0.94)	<0.001		
Platelets count <50,000/dl	13 (4.2)	11 (1.3)	3.54 (1.57–8.00)	0.002		
Prothrombine time <30 %	26 (8.4)	12 (1.5)	6.73 (3.35–13.53)	<0.001	3.02 (1.22–7.49)	0.02
Creatinine level >250 µmol/l	54 (17.5)	70 (9.3)	2.57 (1.75–3.78)	<0.001		
Jaundice	19 (6.2)	17 (8.5)	3.20 (1.64–6.25)	0.0006		
Mechanical ventilation (all setting)	159 (51.6)	316 (38.4)	1.76 (1.35–2.30)	<0.001		
NIV	17 (5.5)	52 (6.3)	0.88 (0.50–1.56)	0.67		
Oxygen administration > 10L/min	76 (24.7)	137 (16.6)	1.68 (1.22–2.32)	0.001		
Preexisting diseases						
COPD	23 (7.5)	45 (5.5)	1.45 (0.86–2.44)	0.16		
Cardiac insufficiency	26 (8.4)	29 (3.5)	2.65 (1.53–4.57)	0.0005		
Evolutive malignancy	54 (17.5)	93 (11.3)	1.74 (1.20–2.50)	0.003		
Cirrhosis	32 (10.4)	24 (2.9)	4.01 (2.32–6.93)	<0.001		
Renal failure with ERT	13 (4.2)	14 (1.7)	2.62 (1.22–5.64)	0.01		
Mac cabe score						
0	90 (31.0)	481 (59.6)	1		1	
1	116 (40.0)	247 (30.6)	2.51 (1.83–3.44)	<0.001	1.64 (1.10–2.46)	0.02
2	84 (29.0)	79 (9.8)	5.68 (3.88–8.32)	<0.001	3.66 (2.23–6.00)	<0.001
SAPS2	60 ± 20	33 ± 15	1.08 (1.07–1.10)	<0.001	1.08 (1.06–1.09)	<0.001

*NIV* non invasive ventilation, *ERT* extra renal therapy

## Discussion

This study showed differences in patient profiles between ICUs with high versus LBA, with no significant difference in patient mortality. Patients admitted to units that usually had available beds were younger and more often had low or high acute illness severity, compared to patients admitted to units with scarce beds.

The ideal ratio of ICU beds to population is difficult to determine [18]. Several studies documented considerable variation in this ratio, both across countries [2] and within countries [3, 4]. An insufficient number of ICU beds results in refusal of patients likely to benefit and therefore in potentially avoidable deaths [17]. Conversely, HBA can result in the admission of patients who are too sick or too well to benefit, thus resulting in the squandering of valuable healthcare resources. In addition to the criteria used for ICU admission decisions, the ideal number of ICU beds is influenced by discharge policies (e.g., the discharge of dying patients to wards and the degree of illness severity at discharge), bed availability in regular wards, and factors influencing ICU stay duration such as weaning or sedation protocols [19, 20].

Previous studies based on questionnaires or case-vignettes strongly suggest that a low number of available ICU beds may influence triage strategies [8, 9]. In studies of everyday practice, the influence of bed availability on triage varied. In a university ICU in Hong Kong with 634 referrals and 236 refusals, bed availability was not significantly associated with admission decisions [21], whereas in several other studies the number of admissions was lower when bed availability was low [10, 11, 13, 14]. In a French ICU, during times without available beds the proportion of patients refused because they were deemed too sick to benefit was larger than the proportion of patients given treatment-limitation decisions after ICU admission (12 versus 1.4 %, *p* < 0.001) [16]. In a study from Canada, among patients refused ICU admission, the proportion deemed too sick to benefit (i.e., requiring palliative care) was larger when a single bed was available than when several beds were available (14.9 versus 8.5 %) [12]. Consistently, data obtained in our study showed that patients with high acute-illness severity were more often refused by units with a shortage of beds.

All these results come together to underline that the identification of patients falling within palliative care abilities is influenced by subjective factors highlighted by shortage of beds. An important result of our study is that high level of bed availability may also influence the decision making process. Apparently, there was no difference with the age or SAPS2 in the admitted population in both groups. But, interestingly, when we categorized the SAPSII values into three groups, we found higher proportions of patients at both ends of the severity spectrum in the HBA group than in the LBA group. This finding suggests that intensivists in HBA units may be more prone to admit either patients who may be too sick or too well to benefit or patients. Very old age has been reported to be associated with denied of ICU admission [22, 23]. In our study, old patients were not refused more often in LBA units, and neither were they admitted more often in HBA units. ICU, day-28, and day-60 mortality rates were not significantly different between HBA and LBA groups. However, HBA units admitted higher proportions of patients at both ends of the severity spectrum, and increased mortality in the sickest patients may therefore have been canceled out by decreased mortality in the patients with less severe illness. When beds are available, intensivists may be more likely to admit more patients even though they are very sick patients for whom futility is a possibility, or younger patients with low illness severity. Thus, the appropriateness of these ICU-admissions is questionable. Several reasons to explain (justify) inappropriate ICU-admission have been reported [24]. Economic considerations might also influence admission decisions leading to inappropriate admission in HBA units [24]. ICU refusal due to bed unavailability has been demonstrated to be associated with increased mortality [17]. This increase also may be due to a higher non-admitted percentage of patients considered as requiring palliative care [12].

Our study has several limitations. First, there is no clear and reliable definition of bed availability which may result from multiple factors, including the number of bed available at the time of triage decision, discharge policies (e.g., the discharge of dying patients to wards and the degree of illness severity at discharge), bed availability in regular wards, and factors influencing ICU stay duration such as weaning or sedation protocols. Thus, since our aim was to conduct a global analysis of triage instead of analyzing admission decisions based solely on the number of beds available on each day, we chose to define bed availability using rates of refusal rather than the number of beds available at the time of triage decision. Second, we can not exclude differences according to centers. Indeed, we did not factor in the ratio of ICU beds in each geographic area over the population in that area. Moreover, although triage decisions are directed by published guidelines [5, 6], we did not determine the extent to which intensivists in each ICU complied with those guidelines. Third, our study was not powered to assess a significant effect of bed availability on mortality. Additionally, policy on decision to forgo life-sustaining therapy may vary in each participating center and may impact ICU mortality. Finally, we did not collect data on the potential effect on admission decisions of non-clinical factors, such as pressure from superiors or economic considerations to use ICU beds more productively.

As expected, the profile of patients admitted and refused in high and low ICU bed availability are different. However, we cannot determine from our data whether HBA units admit patients who are too sick or too well to benefit, or whether LBA units inappropriately refuse patients who are likely to benefit out of concern that their unit would then be unable to admit a patient in greater need of critical care. Further studies are needed to evaluate the impact of bed availability on decisional process for admission of ICU patients.

Key messages:

- Bed availability affects triage decisions.
- Regarding only mean SAPS II and mean age may mask some differences related to the ICU admission of too well or too sick patients.
- The ideal ICU bed/population ratio is a crucial issue for intensivists and administrators.
- A global analysis of triage should be performed instead of analyzing admission decisions based solely on the number of beds available.

## Abbreviations

HBA: high bed availability
LBA: low bed availability
ICU: intensive care unit
OR: odd ratio
SAPS: simplified acute physiologic score
